# Moxibustion for the treatment of Alzheimer's disease: A protocol for a systematic reviews and meta-analysis

**DOI:** 10.1097/MD.0000000000024657

**Published:** 2021-02-12

**Authors:** Rigun A, Ruizhen Yue, Baoshan Chen, Xianbao Huang

**Affiliations:** a Jiangxi University of Traditional Chinese Medicine; b Affiliated Hospital of Jiangxi University of Traditional Chinese Medicine, Nanchang, China.

**Keywords:** Alzheimer's disease, moxibustion, protocol, systematic review

## Abstract

**Background:**

Alzheimer's disease (AD) occurs in the elderly and the early stage of aging, with early clinical manifestations of memory impairment, cognitive impairment, behavioral change and decline in language function, etc., and eventually loss of the ability to live independently, requiring 24-hour care, and a variety of complications. However, these complications are the direct cause of death in AD patients. With the acceleration of the aging process of society, the incidence of AD is increasing year by year, seriously threatening the physical health and quality of life of the elderly. There are many ways to treat AD, however, moxibustion is especially popular in China. Therefore, our systematic review aims to evaluate the efficacy and safety of moxibustion in the treatment of ADand to provide reliable evidence for clinical decision-makers.

**Methods:**

We will search electronic databases including PubMed, Embase, Cochrane Library, China Biomedical Literature Database (CBM), China National Knowledge Infrastructure (CNKI), Wanfang Database (WF), and China Scientific Journals Database (VIP) from inception to January 2021. Two authors will independently screen the studies, extract data information, and assess methodological quality through the Cochrane risk of bias (ROB) tool. The RevmanV.5.3 software will be used for statistical analysis.

**Results:**

The results of this study will evaluate the current status of moxibustion therapy for AD, aiming to prove the effectiveness and safety of moxibustion therapy, and will be published in a peer-reviewed journal.

**Conclusion:**

This systematic review will provide a credible evidence-based for moxibustion in the treatment of AD.

**INPLASY Registration number:**

INPLASY202110021

## 1 Introduction

Alzheimer's disease (AD), also known as “senile dementia”, occurs in the elderly and the early stage of aging, with early clinical manifestations of memory impairment, cognitive impairment, behavioral change and decline in language function, etc.^[1]^ However, studies showed that Aβ deposition is starting before the expected age of dementia onset at −18.9 (SD 3.3) years first, followed by hypometabolism (−14.1 years, SD 5.1), and lastly structural declines (−4.7 years, SD 4.2),^[2]^ and affected people often did not notice these changes in the brain. Over time, the symptoms become more severe, eventually leading to a loss of independence, requiring 24-hour care, and multiple complications such as malnutrition, bedsores, deep vein thrombosis, and infections, however, these complications are the direct cause of death in AD patients.^[3]^ The main pathological changes of AD were hyperphosphorylated Tau agglutination into neurofibrillary tangles,^[4]^ amyloid-βdeposition into senile plaques,^[5]^ and neuronal apoptosis. The etiological factor of AD is not clear, which may be related to age, gender, heredity, lifestyle, low education level, head injury and cardiovascular risk factors.^[6]^ With the accelerated aging process in society, the incidence rate of AD is increasing year by year, which seriously affects the daily life of the elderly and brings heavy burden to the family and the medical care system.^[7]^ In addition, the current commonly used drugs for the treatment of AD cannot reverse the cognitive decline of AD patients, but only to control the symptoms,^[8]^ which are often accompanied by some adverse reactions.^[9,10]^

Moxibustion therapy is an important part of Traditional Chinese medicine and has been practiced in China for thousands of years. Moxibustion is a treatment that uses moxa and other moxibustion materials to burn or warm iron on the body surface, and stimulates acupoints through various functions such as acupoints, drugs and warm stimulation, so as to prevent or treat diseases. Modern researches have shown that the mechanism of moxibustion may be a combination of various factors, such as thermal effect, radiation effect, medicinal properties of moxa, combustion products of moxa and special functions of acupoints.^[11]^ Furthermore, the theory of traditional Chinese medicine believes that moxibustion has the effects of warming Yang and invigorating qi, eliminating phlegm and collateralization, promoting blood circulation and removing blood stasis.^[12]^ It is a traditional external treatment with simple operation, reliable curative effect, and safety.^[13,14]^

Recently, moxibustion can be used not only as the main treatment, but also as a supplementary treatment in combination with drugs to treat AD.^[15,16]^ Related clinical RCTs have proved that moxibustion can effectively alleviate the development process of AD by improving the cognitive function, mental state and daily self-care ability of AD patients, with definite curative and endurable effect.^[17]^ However, there is still a lack of systematic review and meta-analysis on the efficacy and safety of moxibustion in the treatment of AD. Therefore, we will conduct a systematic review and meta-analysis of the efficacy and safety of clinical RCTs of moxibustion in the treatment of AD, with a view to providing reliable evidence for clinical decision makers.

## 2 Methods

### 2.1 Inclusion criteria

#### 2.1.1 Types of studies

RCTs assessing moxibustion treatment for AD will be eligible for inclusion and were published in English or Chinese, with the full-text available.

#### 2.1.2 Types of participants

AD patients with definite diagnostic criteria will be included. The study was not limited to the race, age, gender, nationality and source of cases. Participants who are not suitable for moxibustion will be excluded.

#### 2.1.3 Types of interventions

Moxibustion therapy as a single intervention will include all types of moxibustion (e.g., direct moxibustion, indirect moxibustion, heat-sensitive moxibustion, etc.), or combined with other intervention (e.g., conventional drugs, Chinese herbs, acupuncture, etc.).

#### 2.1.4 Types of control group

There is no limit to the treatment of the control group, including conventional drugs, no treatment, or placebo. RCTs which compare different types of moxibustion therapies/different acupoints will be excluded from our study.

#### 2.1.5 Types of outcome measures

##### 2.1.5.1 Primary outcome

Primary outcomes mainly include the following aspects:

1. Mini-mental state examination (MMSE).
2. Activity of daily living scale (ADL).
3. Alzheimer disease assessment scale-cog (ADAS-cog).
4. Montreal cognitive assessment (MoCA).

##### 2.1.5.2 Additional outcomes

The adverse events will be regarded as an additional result.

### 2.2 Search methods for the identification of studies

We will search electronic databases including PubMed, Embase, Cochrane Library, China Biomedical Literature Database (CBM), China National Knowledge Infrastructure (CNKI), Wanfang Database (WF), and China Scientific Journals Database (VIP) to collect potential RCTs from inception to January 2021. Search terms of disease: senile dementia, AD, presenile dementia, Alzheimer syndrome, Alzheimer sclerosis; and intervention: moxibustion, moxa, suspended moxibustion, mild moxibustion, needle warming moxibustion, thunder fire needle, thunder fire god moxibustion, wheat-sized moxibustion, ginger partitioned moxibustion, governor vessel moxibustion; and research type: randomized controlled trial, controlled clinical trial, randomized. The PubMed search strategy is shown in Table 1.

**Table 1:** Search strategy (PubMed database).

Number	Search items
#1	Mesh: “Alzheimer's disease”
#2	Ti/Ab: “Alzheimer's disease” OR “senile dementia” OR “presenile dementia” OR “Alzheimer syndrome” OR “Alzheimer sclerosis”
#3	#1 OR #2
#4	Mesh: “moxibustion”
#5	Ti/Ab: “moxibustion” OR “moxa” OR “suspended moxibustion” OR “mild moxibustion” OR “needle warming moxibustion” OR “thunder fire needle” OR “thunder fire god moxibustion” OR “wheat-sized moxibustion” OR “ginger partitioned moxibustion” OR “governor vessel moxibustion”
#6	#4 OR #5
#7	Mesh: “randomized controlled trial [Publication Type]” OR “randomized controlled trials as topic” OR “controlled clinical trial [Publication Type]” OR “controlled clinical trials as topic”
#8	Ti/Ab: “randomized”
#9	#7 OR #8
#10	#3 AND #6 AND #9

### 2.3 Data collection and analysis

#### 2.3.1 Selection of studies

**Figure 1:**
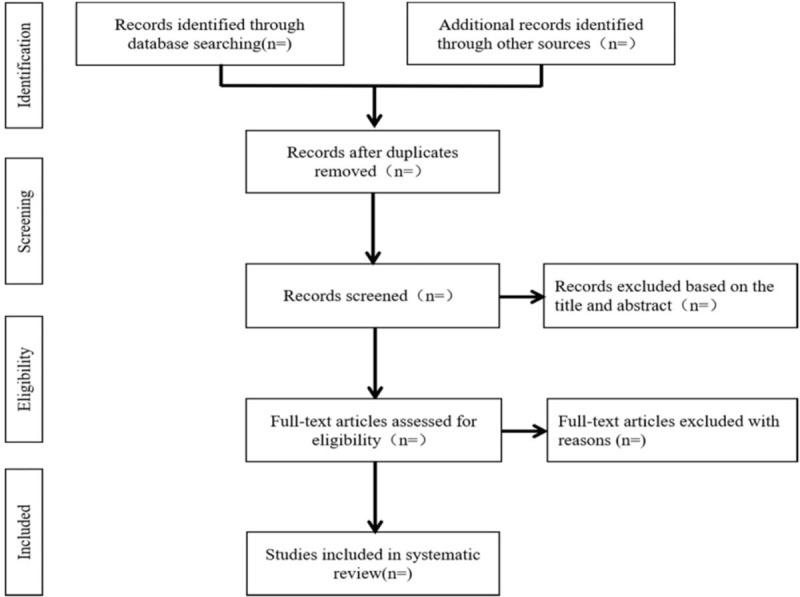
Flow diagram of study selection process.

We will import the retrieved results into EndNote X7 software and delete the duplicate data. After that, two authors will independently scan the titles, abstracts and full texts of the literature according to the inclusion and exclusion criteria to evaluate the eligibility of these articles. Any different opinions will be resolved by the third author. The study selection procedure is summarized in Figure 1.

#### 2.3.2 Data extraction and management

Two reviewers will independently extract relevant data from the eligible RCTs, including the first author, participants’ baseline characteristics, sample size, intervention, intervention time, follow-up, results, and adverse events. Any discrepancies will be resolved through consultation with a third reviewer. If necessary, we will also contact the original author for more information.

### 2.4 Risk of bias assessment

The risk of bias (ROB) assessment tool recommended by Cochrane Collaboration Network was used to evaluate the quality of the included studies. Including the following 7 evaluation items:

(1) random sequence generation;
(2) allocation concealment;
(3) blinding of participants and personnel;
(4) blinding of outcome assessment;
(5) incomplete outcome data;
(6) selective reporting;
(7) other sources of bias.

For each study, the results were rated as “Yes” (low risk), “No” (high risk) and “unclear” (lack of relevant information or uncertainty about bias) for above seven items. Two reviewers independently performed quality assessment and all disagreements will be resolved by discussion.

### 2.5 Quantitative data synthesis and statistical methods

#### 2.5.1 Quantitative data synthesis

We will use RevMan V.5.3 software for statistical analysis. For continuous variables, when outcomes were measured by the same scale, the results were reported as standardized mean difference (MD) and 95% confidence interval (CI); when different scales were used, the results were reported as standardized mean difference (SMD) and 95% CI. Categorical data will be calculated with the risk ratio (RR) and 95% CI.

#### 2.5.2 Assessment of heterogeneity

We will use I^2^ test and Chi-square test to evaluate the heterogeneity of the results. When I^2^ ≤ 50% and *P* > .10, the results of the study will be considered as homogeneous, and fixed effect model will be used; otherwise, random effect model will be used.

#### 2.5.3 Subgroup analysis

If significant heterogeneity is detected in our meta-analysis, we will perform subgroup analysis based on different control groups.

#### 2.5.4 Sensitivity analysis

When there are sufficient RCTs, we will conduct sensitivity analysis based on methodological quality, sample size, and missing data to evaluate the robustness of the research results.

#### 2.5.5 Assessment of reporting biases

Publication bias will be analyzed through the funnel plot. If the funnel plot is asymmetric, there may be a publication bias in the research results.

## 3 Discussion

This study will be a systematic review and meta-analysis on efficacy and safety of moxibustion in treatment for AD. As far as we know, this will be the first study in this field. The conclusion of this study can provide evidence-based medical advice for treating AD with moxibustion, and provide more and better treatment options for AD patients. However, there may be some potential limitations to our conclusions. First, different types of moxibustion, acupoint selection, treatment frequency, treatment duration, and different control groups may lead to potential heterogeneity. Heterogeneity must be explained by subgroup analysis or sensitivity analysis. Second, the measurements and tools for the results of the included randomized controlled trials may differ.

## Author contributions

**Data curation:** Rigun A, Ruizhen Yue.

**Formal analysis:** Rigun A, Ruizhen Yue.

**Investigation:** Rigun A, Ruizhen Yue.

**Methodology:** Baoshan Chen, Ruizhen Yue.

**Project administration:** Rigun A, Xianbao Huang.

**Software:** Ruizhen Yue, Baoshan Chen.

**Supervision:** Xianbao Huang.

**Validation:** Xianbao Huang.

**Writing – original draft:** Rigun A, Xianbao Huang.

**Writing – review & editing:** Rigun A, Xianbao Huang.

## Abbreviations

Abbreviations: 95% CI = 95% confidence interval, AD = Alzheimer's disease, RCTs = randomized controlled trials, ROB = risk of bias.
